# Real-world data analysis of next-generation sequencing and corresponding clinical characteristics in thyroid tumor

**DOI:** 10.1530/EC-24-0301

**Published:** 2024-10-09

**Authors:** Xu-Feng Chen, Cong He, Peng-Cheng Yu, Wei-Dong Ye, Pei-Zheng Han, Jia-Qian Hu, Yu-Long Wang

**Affiliations:** 1 Department of Head and Neck Surgery, Fudan University Shanghai Cancer Center, Shanghai, People’s Republic of China; 2 Department of Oncology, Shanghai Medical College, Fudan University, Shanghai, People’s Republic of China

**Keywords:** thyroid tumor, next-generation sequencing, *BRAF*, *RET*, *TERT* promoter

## Abstract

Next-generation sequencing (NGS) is of great benefit to clinical practice in terms of identifying genetic alterations. This study aims to clarify the gene background and its influence on thyroid tumors in the Chinese population. NGS data and corresponding clinicopathological features (sex, age, tumor size, extrathyroidal invasion, metastasis, multifocality, and TNM stage) were collected and analyzed retrospectively from 2844 individual thyroid tumor samples from July 2021 to August 2022. Among the cohort, 2337 (82%) cases possess genetic alterations, including *BRAF* (71%), *RAS* (4%), *RET/PTC* (4%), *TERT* (3%), *RET* (2.2%), and *TP53* (1.4%). Diagnostic sensitivity before surgery can be significantly increased from 0.76 to 0.91 when cytology is supplemented by NGS. Our results show that *BRAF-*positive papillary thyroid cancer (PTC) patients tend to have older age, smaller tumor size, less vascular invasion, more frequent tumor multifocality, and a significantly higher cervical lymph node metastatic rate. Mutation at *RET* gene codons 918 and 634 is strongly correlated with medullary thyroid cancer. However, it did not display more invasive clinical characteristics. *TERT*-positive patients are more likely to have older age, and have larger tumor size, more tumor invasiveness, and more advanced TNM stage, indicating a poor prognosis. Patients with *TERT, RET/PTC1,* and *CHEK2* mutations are more susceptible to lateral lymph node metastasis. In conclusion, NGS can be a useful tool that provides practical gene evidence in the process of diagnosis and treatment in thyroid tumors.

## Introduction

Next-generation sequencing (NGS) technology is capable of screening hundreds of suspected genes simultaneously using targeted sequencing panels (1). It has been widely used in the detection of high-risk genetic alterations in clinical practice. NGS reveals the comprehensive genetic background of a certain tumor, thus assisting tumor diagnosis and treatment.

Thyroid cancer is the most common endocrine malignancy with an increasing incidence (2) in recent decades. Exciting advances have been made regarding the pathogenesis of thyroid cancer. The *BRAF*^V600E^ mutation, *RAS* mutation, *RET* mutation, *RET*/*PTC* gene translocation, and *TERT* promoter mutation have been screened out as frequently changed genes in various thyroid malignancies (3, 4). To identify and understand the biomarkers of cancer susceptibility is of great clinical value. NGS is a means of learning what is happening behind each thyroid tumor.

NGS panels targeting thyroid diseases, like Thyroseq and Nexthyro, are designed and utilized for both clinical and research purposes (5, 6). Numerous NGS-related studies have been conducted ever since to help better understand thyroid tumors. However, the majority of these studies are carried out with small sample sizes using specific subtypes of thyroid cancer instead of all types of thyroid tumors. Therefore, a large-scale NGS study among all thyroid tumors is required to define its value regarding diagnosis and treatment in clinical practice (7).

Extensive research has elucidated that papillary thyroid cancer (PTC) most frequently harbors the *BRAF^V600E^* mutation (8, 9). When the *TERT* promoter mutation coexists with *BRAF^V600E^*, the thyroid tumor tends to behave more aggressively (10). However, our current understanding of the genetics of thyroid tumors is far from satisfactory. To the best of our knowledge, the prognostic value of *BRAF^V600E^* in thyroid cancer has not been clearly stated yet (11, 12, 13). As for somatic RET mutation, although it has been considered a negative prognostic indicator, some argue that it does not portend compromised DSS or OS in a cohort of medullary thyroid cancer (MTC) patients (14, 15, 16). Additionally, the diagnostic role of NGS in indeterminate thyroid nodules is still controversial (17, 18, 19, 20). In order to quest for further evidence, a large-scale NGS study is needed to describe the genetic background and its influence on thyroid tumors in the Chinese population.

In this paper, we retrospectively collected and analyzed NGS data along with corresponding clinical information from thyroid tumor patients at our institute. Genetic alterations are noted and compared in both thyroid fine-needle aspiration (FNA) samples and surgical specimens. Our study provides a thorough understanding of common genetic changes and their clinical significance in thyroid tumors.

## Materials and methods

### Data collection

We collected 2844 consecutive thyroid NGS testing reports from July 2021 to August 2022 at Fudan University Shanghai Cancer Center. NGS analysis was conducted using either preoperative FNA samples or surgical specimens. The type of tissue (thyroid tumor, metastatic lymph node, or other metastatic tissue) used in the analysis was recorded.

Corresponding clinical information (sex, age, pathology, tumor size, multifocality, extrathyroidal invasion, metastasis, and Tumor, Node, Metastasis (TNM) stage) was retrieved from the electronic medical record system. All samples (including FNA and surgical specimens) in our study were reviewed separately by at least two experienced pathologists to produce the final pathology report. TNM stage was assessed according to the 8th edition of the AJCC/UICC TNM staging system. The study was reviewed and approved by the Ethics Committee of Fudan University Shanghai Cancer Center. Patients/participants provided their written informed consent before the study.

### NGS sequencing

The NGS test is based on the Illumina high-throughput sequencing platform and uses liquid-phase capture technology to detect DNA mutations. The average sequencing depth of DNA single-nucleotide variation and small fragment insertion/deletion is >1000×. The lower limit of mutation detection frequency is 1–2%, and the lower limit of gene fusion detection is also 1–2%.

The sequence reference genome is the human genome UCSC hg19 February 2009, and the variable naming rules refer to HGVS.

FNA, along with BRAF single-gene detection, is routine for patients with thyroid tumors at our institute. NGS analysis is a complementary test and is considered under the following circumstances: i) when a patient is suspected to have thyroid malignancy other than PTC; ii) indeterminate nodules with undetermined or negative *BRAF* results; and iii) patients with end-stage tumors or aggressive clinical manifestations. Three NGS panels are available for thyroid cancer detection at our institute. Data from all three panels were collected in this study. One covers 30 genes, another covers 32 genes, and the third panel covers 88 genes (detailed information is listed in Supplementary Table 1; see section on supplementary materials given at the end of this article). Gene scale and medical expenses are two major decisive factors in choosing different NGS panels. Doctors may prescribe different NGS panels based on medical necessity.

### Statistical analysis

Statistical analysis and correlation tests were performed using SPSS software (version 20.0, IBM). The *t*-test was used for continuous variables such as age and tumor size, while the chi-square test or Fisher's exact test was used for classified variables such as gender and pathological type. *P* values <0.05 were considered statistically significant. A waterfall diagram of the gene mutation spectrum was generated using oncoprints with the visualization tool from cBioPortal (https://www.cbioportal.org/visualize) (21, 22).

## Results

### Baseline information of NGS samples analyzed in this study

In this study, 2844 consecutive and complete thyroid NGS data analyzed at our institute from July 2021 to August 2022 were collected retrospectively. The corresponding clinical pathological information was retrieved from the electronic medical record system. The baseline information of NGS samples analyzed in this study is listed in Table 1.

**Table 1:** Baseline information of NGS samples analyzed in this study (*n* = 2844).

Clinicopathological parameters	
Sex	
Female	1993 (70.1%)
Male	851 (29.9%)
Age (years)	43.5 ± 12.6
Sample source	
Surgical specimen	2149 (75.5%)
FNA	695 (24.5%)
Sample tissue type	
Thyroid tissue	2650 (93.1%)
Metastatic lymph node	179 (6.3%)
Other metastatic tissue	15 (0.5%)
NGS panel	
30 genes	2045 (71.9%)
32 genes	700 (24.6%)
88 genes	99 (3.5%)
Pathology	
Benign tumor	170 (6.0%)
Uncertain malignant potential	100 (3.5%)
PTC	2425 (85.3%)
FVPTC	19 (0.7%)
FTC	22 (0.8%)
MTC	64 (2.3%)
ATC or PDTC	30 (1.0%)
Other malignancy	14 (0.5%)
Tumor size (mm)	12.9 ± 9.9
Multifocality	745 (26.2%)
Extrathyroidal invasion	
Capsular invasion	111 (4.8%)
Vascular invasion	216 (9.3%)
Nerve invasion	54 (2.3%)
Muscle invasion	61 (2.6%)
TNM	2329
I	2103 (90.3%)
II	148 (6.4%)
III	48 (2.1%)
IV	30 (1.3%)

Among all 2844 patients, 1993 (70.1%) are women. The average age at NGS analysis is 43.5 ± 12.6 years. The majority of NGS samples come from surgical specimens (2194), while the rest (695) are from FNA samples. When it comes to sample tissues, 2650 (93.1%) of the NGS analyses are based on thyroid tumors, 179 (6.3%) are based on metastatic lymph nodes, and the remaining 15 (0.5%) are based on other metastatic tissues, such as lungs and bones.

Our institute provides three types of NGS panels for thyroid cancer analysis, mainly differing in gene scales (30, 32, and 88 genes). In this study, 2045 (71.9%) samples are analyzed using the 30-gene panel, 700 (24.6%) with the 32-gene panel, and 99 (3.5%) with the 88-gene panel.

Of all samples, 170 (6.0%) are benign tumors, 100 (3.5%) are classified as having uncertain malignant potential, and the remaining 2574 are malignant thyroid tumors. Regarding all malignant cases, 2425 of them are PTC, 64 are MTC, 30 are anaplastic thyroid cancer (ATC) or poorly differentiated thyroid cancer (PDTC), 22 are follicular thyroid cancer (FTC), 19 are follicular variant of papillary thyroid cancer (FVPTC), and the remaining 14 are rare tumors, including four thyroid squamous cell carcinoma, two intrathyroid thymic carcinoma (ITC, or ‘CASTLE’ in old terminology), two oncocytic carcinoma (OCA), two carcinosarcoma, one B cell non-Hodgkin lymphoma, and one metastatic lung adenocarcinoma.

In the cohort, 2574 patients were diagnosed with malignant thyroid tumors, and 2329 of them received surgical treatment at our hospital. Postoperative clinical data were collected from patients who underwent surgery. The average tumor size is 12.9 ± 9.9 mm. Multifocality is seen in 745 (26.2%) samples, 216 (9.3%) showed vascular invasion, 54 (2.3%) had nerve (2.3%) invasion, 61 (2.6%) had muscle invasion, and 111 (4.8%) had capsular invasion.

According to the 8th edition of the AJCC/UICC TNM staging system, TNM information can be acquired from 2329 samples. Among those, 2103 (90.3%) are stage I, 148 (6.4%) are stage II, 48 (2.1%) are stage III, and 30 (1.3%) are stage IV.

### Gene mutation status

**Figure 1:**
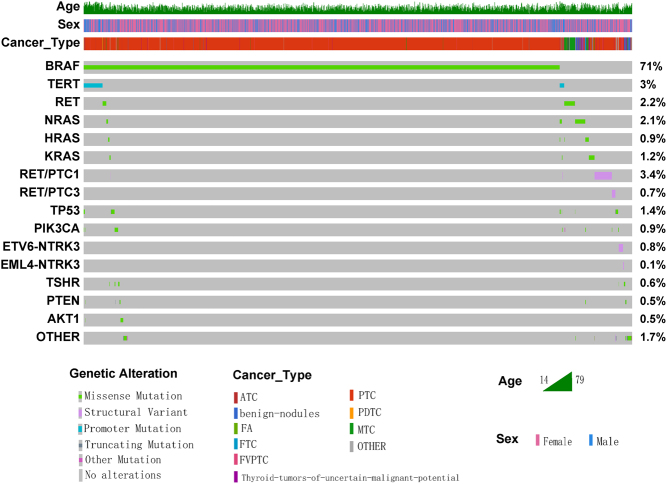
Oncoprint of gene mutations in this study (*n* = 2337). ATC, atypia thyroid carcinoma; FA, follicular adenoma; FTC, follicular thyroid carcinoma; FVPTC, follicular variant of papillary thyroid carcinoma; PTC, papillary thyroid carcinoma; PDTC, poorly differentiated thyroid carcinoma; MTC, medullary thyroid carcinoma; OTHER, genes with a mutation rate < 0.5% were classified as OTHER.

All 2844 NGS samples generated valid gene reports. In the analysis, 2337 (82%) of them had gene changes detected. The occurrence of each gene mutation is shown below (Fig. 1). Results indicate that *BRAF*, *RAS*, *TERT*, *TP53*, *RET* mutation, and *RET/PTC* fusions are the six most common genetic mutations in thyroid tumors. The *BRAF* gene has the highest mutation frequency of 71%. Genes with a mutation frequency of less than 0.5% are classified into ‘OTHER’ in this study (Supplementary Table 2). According to our data, *CHEK2* (*n* = 8), *EZH1* (*n* = 7), and *SPOP* (*n* = 6) (variants see Supplementary Table 4) are the top three mutations in the ‘OTHER’ group.

A total of 2113 cases were detected with single-gene mutations, and 222 cases were detected with multiple-gene co-mutations. The most prevalent multiple-gene pattern is the *BRAF+TERT* co-mutation (36.5%). *BRAF* is also the most common single gene mutated in PTC, with a frequency of 82%. In FTC and FVPTC, the most common genetic mutation is *NRAS* (19.5%). *RET* is the leading genetic mutation in MTC, with a frequency of 59.3%. In PDTC and ATC, the most frequently mutated gene is *TERT* (57.1%).

In this study, genes with a mutation rate <0.5% were classified as rare. Eighteen rare single-gene mutations were detected and analyzed (Table 2). NGS revealed 76* RET/PTC1* and 16 RET*/PTC3* mutant cases in our cohort. Notably, 73 *RET/PTC1* (94.7%) and 15 *RET/PTC3* (93.7%) mutant cases are detected in PTC. As for the *NTRK3* gene, there are three *EML4-NTRK3 (100%)* mutant cases, 16 *ETV6-NTRK3* (84.2%) mutant cases, and one* SQSTM1-NTRK3* (100%) mutant case detected in PTC. These data provide evidence that *RET/PTC* and *NTRK3* fusion genes are highly correlated with PTC, suggesting they may have diagnostic value in clinical practice. On the other hand, *CTNNB1* (1)*, GNAS* (2)*, NTRK1* (1), and *ZNF148* (4) gene mutations occur exclusively in benign tumors with low mutation frequency, indicating they lack the capability to initiate malignant progression on their own.

**Table 2:** Incidence of other rare single gene mutations and pathological tumor types.

Gene alteration	Pathology (%)							
	Benign	Uncertain	PTC	FVPTC	FTC	ATC	PDTC	OTHER
CTNNB1	1 (100)							
CDKN2A							1 (100)	
CHEK2								1 (100)
EIF1AX	2 (66.6)		1 (33.3)					
EML4-NTRK3			3 (100)					
ETV6-NTRK3		1 (5.3)	16 (84.2)	2 (10.5)				
SQSTM1-NTRK3		1 (100)						
EZH1	1 (33.3)	1 (33.3)	1 (33.3)					
GNAS	2 (100)							
NTRK1	1 (100)							
PTEN	4 (80)					1 (20)		
RET/PTC1		3 (3.9)	72 (94.7)	1 (1.3)				
RET/PTC3		1 (6.3)	15 (93.7)					
SPOP	5 (83.3)		1 (16.7)					
TERT			3 (60)		1 (20)	1 (20)		
TP53			5 (55.6)			2 (22.2)	1 (11.1)	1 (11.1)
TSHR	4 (66.6)	1 (16.7)	1 (16.7)					
ZNF148	4 (100)							

### Application of NGS in preoperative diagnosis of thyroid cancer

#### Assisting diagnosis of cytology indeterminate nodules with NGS

Among all NGS samples, 695 of them are from FNA. These samples were all tested by the 30-gene NGS panel, therefore suitable for analysis. Among these cases, 42 are Bethesda category 1 nodules, 134 are Bethesda category 2 nodules, 114 are Bethesda category 3 or 4 nodules, and 391 are Bethesda category 5 or 6 nodules.

Among the 695 NGS cases derived from FNA, 308 patients received thyroidectomy at our hospital. Before surgery, 231 of these cases had malignant cytology results, and 77 nodules had indeterminate cytology results. An indeterminate tumor is considered malignant if NGS results reveal *BRAF* or *TERT* mutations. Additionally, indeterminate nodules with gene mutations other than CTNNB1, GNAS, NTRK1, and ZNF148 (which exclusively occur in benign tumors as described in Table 2) are considered malignant in this study. To determine the diagnostic efficiency of NGS in FNA nodules, we compare the sensitivity and specificity between cytological diagnosis alone and the combination of cytology plus NGS (Table 3). The postoperative pathology report was acquired and served as the ‘golden standard’ in this analysis.

**Table 3:** Diagnostic efficiency of cytology and cytology combined with NGS (*n* = 308).

Postoperative pathology report	Cytology				
	Malignant	Indefinite			
Malignant	231	71	Se 0.76	Sp 1	YI 0.76
Benign	0	6	PPV 1		NPV 0.08
**Postoperative pathology report**	**NGS plus cytology**				
	Cytology malignant or (with) pathogenic mutation	Cytology indefinite without pathogenic mutation			
Malignant	277	25	Se 0.91	Sp 0.33	YI 0.25
Benign	4	2				PPV 0.99	NPV 0.07	

NPV, negative predictive value; PPV, positive predictive value; Se, sensitivity; Sp, specificity; YI, Youden index.

In those 77 cytology indeterminate cases, postoperative pathology identified 71 malignant and six benign tumors. With the help of NGS testing, 46 indeterminate cases were diagnosed before surgery. Notably, four cases were false positives in the NGS results. These nodules were considered benign according to postoperative pathology reports. The detailed gene variants are *KRAS, NRAS, EIF1AX,* and* BRAF*. The combination of cytology and NGS data significantly increased diagnostic sensitivity from 0.76 to 0.91, with an obvious decrease in specificity. However, due to sample bias (Bethesda 1–4 samples are often not subjected to NGS testing or surgical treatment), the sample size of pathology-confirmed benign tumors is extremely small. Besides, postoperative samples were not tested by NGS in this study. There is no information on whether NGS analysis of FNA samples and postoperative samples was consistent in terms of the gene variants present. Our results showed low specificity (0.33), negative predictive value (0.07), and Jordan index (0.25).

According to our findings, NGS can be a useful preoperative diagnostic approach in addition to cytology. The result of NGS should be read with caution to avoid false positive conclusions.

#### Correlation of RET gene mutation site and thyroid tumor pathology type

**Figure 2:**
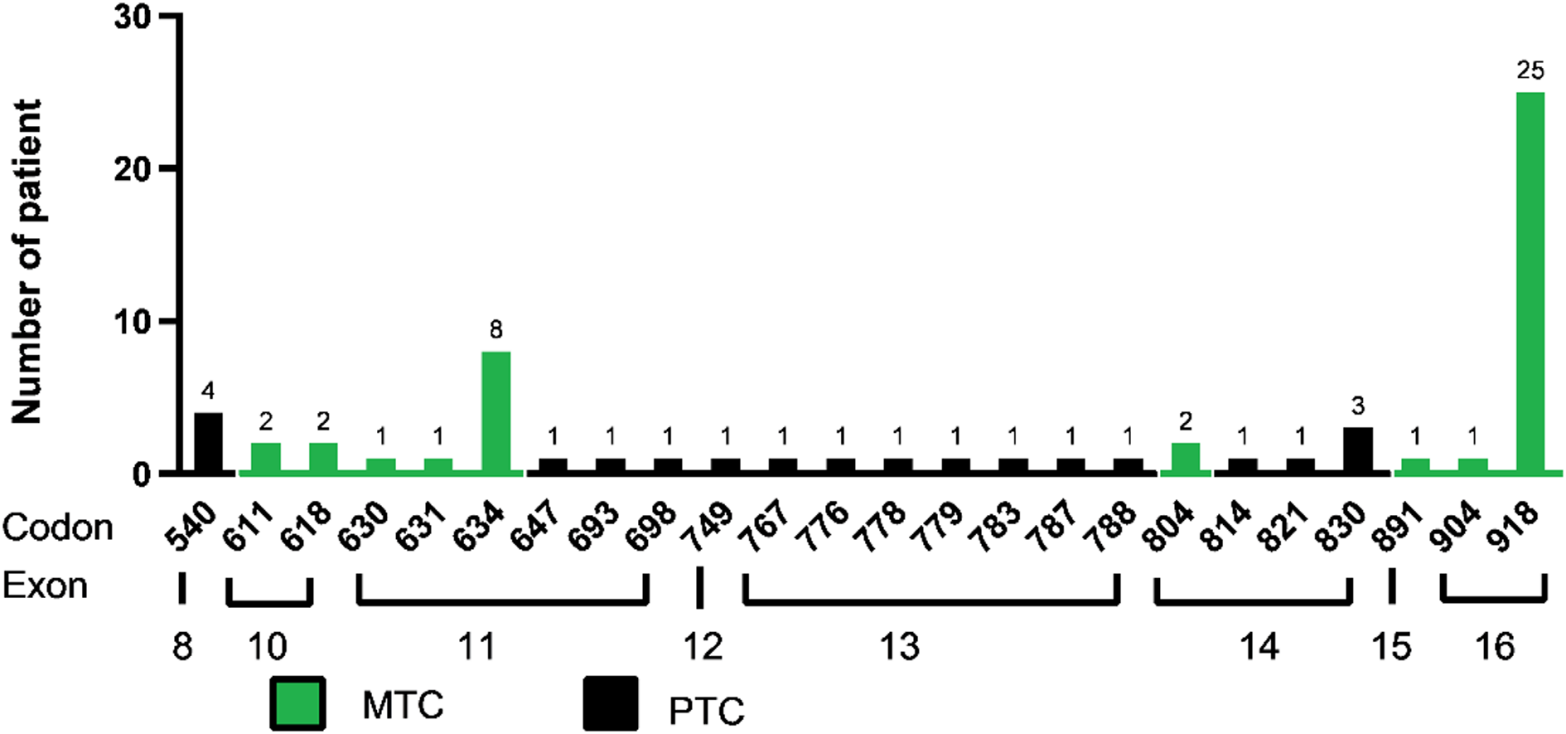
Frequency diagram of mutation sites of RET gene. The column height represents the number of cases where codon mutation at this site is detected, and the green part represents the pathological diagnosis of MTC.

Among all 2844 samples, 63 exhibit RET gene mutations. We counted the frequency of occurrence of various mutation sites in the *RET* gene (Fig. 2). The most common mutation sites are codon 918 (*n* = 25) and codon 634 (*n* = 8). The mutation at codon 918 involves a change from ATG (methionine) to ACG (threonine). Met918 is a key component of the substrate recognition pocket in the catalytic core of the *RET* protein tyrosine kinase (23). Cases with this mutation are mostly MTC, except for one follicular tumor with undetermined malignant potential. Patients with mutations in codon 634 are all MTC.

Regarding *RET* point mutations, 42 cases present point mutations in the *RET* gene alone. The pathology of these tumors includes MTC (*n* = 38), OCA (*n* = 1), PTC (*n* = 1), and atypical follicular epithelium (*n* = 2). The mutation site of oncocytic carcinoma is at codon 693 in exon 11 of c.2077C>T, leading to the Arg to Cys change. There are 21 cases of *RET* point mutation on codon 918 that also harbor other gene variations. *BRAF* is the most commonly co-mutated gene (*n* = 16). Among all the cases of *RET* and *BRAF* co-mutated tumors, 14 cases are PTC (*n* = 14), and the other two are MTC combined with PTC. Detailed *RET* mutations that co-occur with *BRAF* mutations are listed in Supplementary Table 3. Other *RET* co-mutated genes found in this research are *HRAS, KRAS, TP53, TERT*, and *PIK3CA*. All of these tumors are MTC except for one PTC which is RET co-mutated with *TERT*. Our results suggest a strong correlation between *RET* point mutation and MTC.

### Application of NGS in prognosis of thyroid cancer

#### *BRAF* mutation and its correlation with tumor prognosis

*BRAF* mutations were detected in 2026 (71%) of all 2844 analyzed cases, while in all PTC patients, 1985 cases (82%) are *BRAF* positive. The positive rate is higher than those described in previous studies (24), suggesting *BRAF* mutation may have a higher positive rate in the Chinese population.

We further analyzed the clinicopathological features of PTC patients with positive *BRAF* mutation (Table 4). Compared to *BRAF*-negative PTC patients, *BRAF-*positive patients tend to be diagnosed at an older age (*P* = 0.046). *BRAF-*positive PTC tumors are smaller (*P* = 0.014) and show multifocality more often (*P* = 0.049). *BRAF*-positive PTC tumors also exhibit lower vascular invasion and lateral cervical lymph metastasis (*P* < 0.001). As for TNM staging, tumors analyzed in this study showed no significant results regardless of their *BRAF* mutation status.

**Table 4:** Clinical features of *BRAF^V600E^* mutation in PTC patients (*n* = 2425).

	*BRAF*^+^ (%)	*BRAF*^−^ (%)	*P*
Number	1985	440	
Sex			
Male	612 (30.8)	117 (26.6)	0.079
Female	1373 (69.2)	323 (73.4)	0.079
Age (years)	42.84 ± 12.13	41.55 ± 12.72	0.046^a^
Tumor size (mm)	11.98 ± 8.17	13.41 ± 10.97	0.014^a^
Multifocality	602 (34.1)	106 (28.8)	0.049^a^
Extrathyroidal invasion			
Vascular invasion	135 (6.9)	57 (13.5)	<0.001^c^
Nerve invasion	41 (2.1)	5 (1.2)	0.214
Muscle invasion	41 (2.1)	10 (2.4)	0.733
Capsular invasion	76 (3.9)	15 (3.5)	0.737
Lymph node metastasis	1086 (54.7)	272 (61.8)	0.007^b^
Central lymph node metastasis	986 (49.7)	232 (52.7)	0.246
Lateral cervical lymph metastasis	506 (25.5)	180 (40.9)	<0.001^c^
Extranodal invasion	146 (7.8)	43 (10.4)	0.088
TNM			
I	1670 (91.9)	338 (89.2)	0.091
II	103 (5.7)	30 (7.9)	0.095
III	37 (2)	9 (2.4)	0.675
IV	8 (0.4)	2 (0.5)	1.000

^a^*P*< 0.05; ^b^*P*< 0.01; ^c^*P*< 0.001.

The point mutation at base c.1799T>A of *BRAF*^V600E^ is the dominant type in all *BRAF* gene mutations detected (*n* = 2024). It is notable that the discovery of a base c.1801A>G mutation ultimately led to the diagnosis of a dominant follicular tumor with follicular carcinoma with minimal invasion in some areas. The remaining case exhibits a *BRAF* base c.1862A>G mutation combined with a *KRAS* base c.34G>A mutation. The diagnosis is a rare large, poorly differentiated thyroid B-cell non-Hodgkin's lymphoma.

There are 187 cases possessing the *BRAF* mutation along with other mutations, and the specific co-mutations are shown in Fig. 1. The most common is the TERT promoter mutation. Of all 81 co-mutant cases, 71 (87.7%) show more aggressive PTC, which is consistent with the existing research conclusions (25). Notably, there are ten *BRAF* mutant tumors with the pathological diagnosis of undifferentiated or poorly differentiated cancer. Except for one case where *BRAF* was combined with the* AKT1* mutation, the remaining nine cases were all *BRAF* combined with the *TERT* promoter mutation. Tumors with both *BRAF* and *TERT* co-mutations are often combined with *TP53* (*n* = 3) or *PIK3CA* (*n* = 3) three-gene co-mutation or four-gene co-mutation (*n* = 1). These results suggest that a *BRAF* mutation alone may not be sufficient to cause the dedifferentiation process in thyroid cancer.

#### *RET* mutation and its correlation with MTC prognosis

*RET* point mutations were found in 63 (2.2%) of all NGS samples. Among the detected MTC samples, 42 cases (66.7%) were positive for *RET* point mutations. There is clear evidence that germline missense mutations in the *RET* proto-oncogene are essential for the occurrence and development of familial FMTC, while somatic *RET* mutations that occur later in life may lead to sporadic MTC (23, 26). Unfortunately, in this study, we are not able to tell if the *RET* mutation is somatic or germline. We compared the clinicopathological features between MTC patients with and without *RET* point mutation based on our cohort (Table 5). The analysis did not show any statistically significant clinical features regarding extrathyroidal invasion, lymph node metastasis, or TNM status.

**Table 5:** Clinical features of MTC patients (*n* = 64).

	*RET*^+^ (%)	*RET*^−^ (%)	*P*
Number	42	22	
Sex			
Male	15 (35.7)	10 (45.5)	0.448
Female	27 (64.3)	12 (54.5)	0.448
Age (years)	49.3 ± 13.1	51.6 ± 10.6	0.494
Tumor size (mm)	16.0 ± 11.8	14.9 ± 10.4	0.778
Multifocality	10 (43.5)	5 (45.5)	1.000
Extrathyroidal invasion			
Vascular invasion	7 (19.4)	2 (12.5)	0.831
Nerve invasion	0 (0.0)	1 (6.2)	0.674
Muscle invasion	0 (0.0)	0 (0.0)	1.000
Capsular invasion	0 (0.0)	1 (6.1)	0.674
Lymph node metastasis	38 (90.5)	18 (81.8)	0.551
Central lymph node metastasis	23 (54.8)	8 (36.4)	0.162
Lateral cervical lymph metastasis	18 (42.9)	6 (27.3)	0.221
Extranodal invasion	5 (13.2)	2 (11.1)	0.829
TNM			
I	20 (69.0)	9 (75.0)	0.993
II	7 (24.1)	3 (25)	1.000
III	0 (0)	0 (0)	1.000
IV	2 (6.9)	0 (0)	1.000

#### *TERT* promoter mutation indicates poor prognosis in thyroid tumors

In this study, the mutation in the *TERT* promoter is detected in 99 samples (3%). Compared to* TERT-*negative patients, patients with *TERT* mutations were diagnosed at an older age (58.9 ± 12.7 vs 42.9 ± 12.2 yrs, *P* < 0.001). *TERT-*positive tumors are much bigger (25.4 ± 16.3 vs 12.6 ± 9.5 mm, *P* < 0.001) and tend to exhibit more extrathyroidal invasion. Moreover, *TERT* mutations are strongly associated with ATC and show extremely poor prognostic features, leading to more advanced TNM staging (Table 6).

**Table 6:** Clinical features of patients with or without *TERT* mutation.

	*TERT^+^*(%)	*TERT^−^* (%)	*P*
Number	99	2745	
Sex			
Male	50 (50.5)	801 (29.2)	<0.001^c^
Female	49 (49.5)	1944 (70.8)	<0.001^c^
Age (years)	58.9 ± 12.7	42.9 ± 12.2	<0.001^c^
Tumor size (mm)	25.4 ± 16.3	12.6 ± 9.5	<0.001^c^
Multifocality	20 (46.5)	725 (32.6)	0.054
Extrathyroidal invasion			
Vascular invasion	13 (15.9)	202 (7.6)	0.006^b^
Nerve invasion	5 (6.1)	47 (1.8)	0.015^a^
Muscle invasion	11 (13.6)	50 (1.9)	<0.001^c^
Capsular invasion	12 (14.8)	86 (3.2)	<0.001^c^
Lymph node metastasis	49 (49.5)	1329 (48.4)	0.833
Central lymph node metastasis	35 (35.4)	1190 (43.4)	0.114
Lateral cervical lymph metastasis	40 (40.4)	690 (25.14)	0.001^b^
Extranodal invasion	13 (22.0)	189 (8.0)	<0.001^c^
Pathology			
Benign	0 (0)	170 (6.2)	<0.001^c^
PTC	76 (76.8)	2349 (85.6)	0.015^a^
FVPTC	2 (2.0)	17 (0.6)	0.292
PDTC	3 (3.0)	6 (0.2)	<0.001^c^
FTC	3 (3.0)	19 (0.7)	0.043^a^
MTC	0 (0)	64 (2.3)	0.233
ATC	14 (14.1)	7 (0.3)	<0.001^c^
Other malignancy	1 (1.0)	13 (0.5)	0.985
Uncertain malignancy	0 (0)	100 (3.6)	0.098
TNM			
I	23 (41.8)	2079 (91.6)	<0.001^c^
II	15 (27.3)	133 (5.9)	<0.001^c^
III	8 (14.5)	38 (1.7)	<0.001^c^
IV	9 (16.4)	20 (0.9)	<0.001^c^

^a^*P*< 0.05; ^b^*P*< 0.01; ^c^*P*< 0.001.

**Figure 3:**
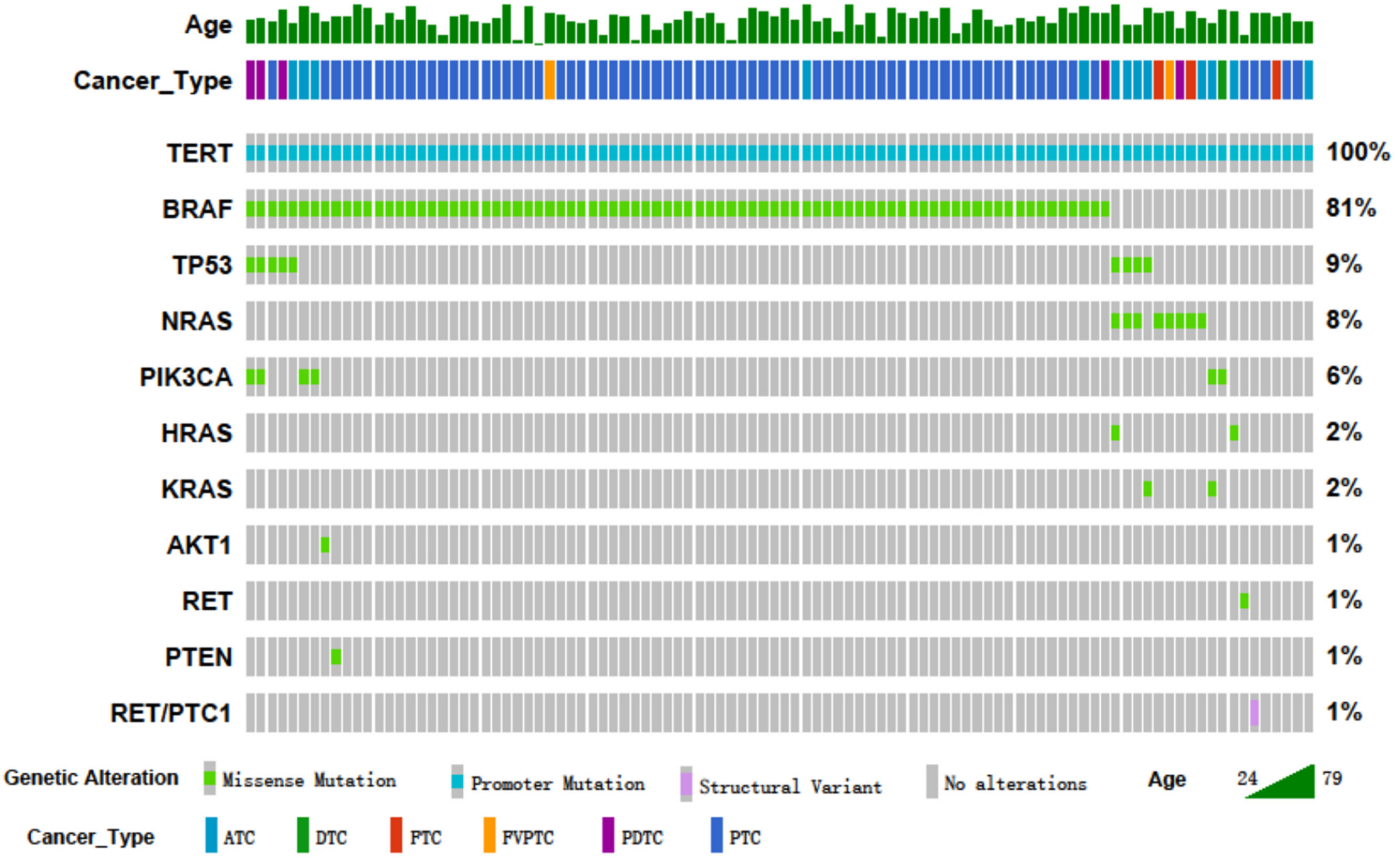
Oncoprint of gene mutation in TERT+ samples. ATC, atypia thyroid carcinoma; DTC, differentiated thyroid carcinoma; FTC, follicular thyroid carcinoma; FVPTC, follicular variant of papillary thyroid carcinoma; PDTC, poorly differentiated thyroid carcinoma; PTC, papillary thyroid carcinoma.

Only five samples had a *TERT* single-gene mutation, which means the other 94 samples are positive for mutation in addition to *TERT*. In fact, 80 cases are double gene co-mutations, 11 cases are triple gene co-mutations, and two cases are quadruple gene co-mutation. The co-mutation information is shown in Fig. 3. FTC, PTC, ATC, and ATC are presented in the five cases with only single-gene mutation of *TERT*. Due to the small sample size, no significant clinical feature is found. It should be noted that *TERT* is often co-present with *TP53* (*n* = 9) and *PIK3CA* (*n* = 5) in *BRAF* (*n* = 7) or *RAS* (*n* = 4) positive cases. Those three-gene co-mutation or four-gene co-mutation tumors are all ATC or spindle cell malignancies, suggesting a possible synergistic effect of *TERT* with *TP53* or *PIK3CA* in tumor dedifferentiation.

#### Gene mutation and lymph node metastasis

Prophylactic dissection of central compartment lymph nodes is routine in the surgical treatment of malignant thyroid tumors (except for FTC) at our institution. We focused on those patients who have negative findings (ultrasound and contrast neck CT) of cervical lymph node metastasis (cN0, *n* = 1239) before surgery. Postoperative pathology reports were analyzed and discovered, 458 of them had central lymph node metastasis (pN1). Detailed NGS information was compared to determine if a certain gene mutation can be predictive for central lymph node metastasis (Table 7).

**Table 7:** Gene mutation and postoperative central lymph node metastasis in cN0 patients (*n* = 1239).

Targted gene	cN0 (%) with targeted gene mutation	pN1 (%) with targeted gene mutation	pN1 (%) without targeted gene mutation	*P*
BRAF	1023 (82.57)	397 (38.81)	61 (28.24)	0.003^b^
KRAS	18 (1.45)	2 (11.11)	456 (37.35)	0.022^a^
RET	22 (1.78)	8 (36.36)	450 (36.98)	0.953
NRAS	18 (1.45)	5 (27.78)	453 (37.10)	0.416
RET/PTC1	15 (1.21)	6 (40.00)	452 (36.93)	0.806
TP53	10 (0.81)	2 (20.00)	456 (37.10)	0.431
PIK3CA	7 (0.56)	1 (14.29)	457 (37.09)	0.393
ETV6-NTRK3	6 (0.48)	2 (33.33)	456 (36.98)	1
HRAS	4 (0.32)	0 (0.00)	458 (37.09)	0.31
TERT	11(0.89)	7 (63.64)	451 (36.73)	0.127
SPOP	4 (0.32)	1 (25.00)	457 (37.00)	1
AKT1	4 (0.32)	1 (25.00)	457 (37.00)	1
RET/PTC3	3 (0.24)	1 (33.33)	457 (36.97)	1
PTEN	2 (0.16)	0 (0.00)	458 (37.03)	0.726
TSHR	6 (0.48)	4 (66.67)	454 (36.82)	0.277
OTHER	8 (0.65)	3 (37.50)	455 (36.96)	0.407
NONE	138 (11.14)	40 (28.99)	418 (37.97)	0.039^a^

^a^*P*< 0.05; ^b^*P*< 0.01.

OTHER, genes with a mutation rate <0.5% were classified as OTHER.

After surgery, 397 (38.81%) presumed cN0 *BRAF* mutant patients were found to have cervical lymph node metastasis, which is significantly higher than those without a *BRAF* mutation (28.24%, *P* = 0.003). Other gene mutations, such as* RET, NRAS, RET/PTC1,* and *TP53*,did not show notable value in predicting central lymph node metastasis. However, patients with no identified gene mutation (*n* = 138) had a significantly lower rate of central lymph node metastasis after surgery compared to those with at least one gene mutation detected (28.99% vs 37.97%, *P* = 0.039). The result of this part should be interpreted with caution due to the influence of the dissection area in reality by different surgeons. Nevertheless, patients with *BRAF* mutations tend to have occult central lymph node metastasis and need to be taken into clinical consideration.

We further explored the correlation between gene mutation and lateral lymph node metastasis in all 2844 patients who received surgery (Table 8). *BRAF* is the most commonly mutated gene (515, 70.55%) in all 730 patients with lateral lymph node metastasis. However, there is no significant difference in lateral lymph node metastatic rate regarding *BRAF* mutation status. Notably, patients with *TERT* (40.40% vs 25.14%, *P* =0.001)*, RET/PTC1* (41.56% vs 25.23%, *P* = 0.001) and *CHEK2* (62.50% vs 25.56%, *P* = 0.047) mutations are more susceptible to lateral lymph node metastasis.

**Table 8:** Gene mutation and postoperative lateral lymph node metastasis (*n* = 2844).

Targeted gene	Case of mutation	pN1 (%) with targeted gene mutation	pN1 (%) without targeted gene mutation	*P*
BRAF	2030	515 (25.37)	215 (26.41)	0.565
TERT	99	40 (40.40)	690 (25.14)	0.001^c^
RET/PTC1	77	32 (41.56)	698 (25.23)	0.001^c^
RET	63	20 (31.75)	710 (25.53)	0.264
NRAS	58	8 (13.79)	722 (25.92)	0.036^a^
TP53	41	13 (31.71)	717 (25.58)	0.373
KRAS	33	4 (12.12)	726 (25.83)	0.073
PIK3CA	26	9 (34.62)	721 (25.59)	0.294
HRAS	25	9 (36.00)	721 (25.58)	0.235
ETV6-NTRK3	20	8 (40.00)	722 (25.57)	0.141
RET/PTC3	16	6 (37.50)	724 (25.60)	0.424
TSHR	16	1 (6.25)	729 (25.78)	0.135
AKT1	13	4 (30.77)	726 (25.64)	0.917
PTEN	13	2 (15.38)	728 (25.72)	0.594
CHEK2	8	5 (62.50)	725 (25.56)	0.047^a^
OTHER	19	9 (47.37)	721 (25.52)	0.056
NONE	508	128 (25.20)	602 (25.77)	0.788

^a^*P* < 0.05; ^c^*P*< 0.001.

OTHER, genes with a mutation rate <0.5% were classified as OTHER.

### NGS information for screening appropriate targeted therapy

Targeted therapy has become a promising approach in the multi-disciplinary treatment of patients with late-stage or recurrent thyroid cancer. NGS information holds substantial clinical significance in guiding personalized targeted treatment approaches. We employed OncoKB (http://oncokb.org/) in our study to identify level 1 targets (FDA-recognized biomarkers predictive of response to an FDA-approved drug) and their corresponding FDA-approved targeted therapies (Table 9). In those with pathologically confirmed malignant thyroid tumors, NGS is able to identify 16 kinds of gene mutations that have mature targeted therapies. Four of these mutations (*BRAF, RET, RET/PTC,* and*NTRK3*) possess targeted therapies that have been approved in thyroid cancers. Although the other 12 genes have not been considered as valid targets yet, their future potential cannot be neglected. NGS is an efficient tool to identify and screen available drugs.

**Table 9:** Gene mutation frequency and corresponding FDA-approved targeted drugs.

	Frequency	Drug
BRAF	2030	Dabrafenib, Trametinib
RET/PTC1	77	Pralsetinib, Selpercatinib
RET	63	Pralsetinib, Selpercatinib
PIK3CA	26	Alpelisib, Fulvestrant
ETV6/NTRK3	19	Entrectinib, Larotrectinib
RET/PTC3	16	Pralsetinib, Selpercatinib
CHEK2	8	Olaparib
EML4/NTRK3	3	Entrectinib, Larotrectinib
ALK	2	Crizotinib, Alectinib
EZR-ROS1	1	Crizotinib, Entrectinib
FGFR1	1	Pemigatinib
FGFR3	1	Erdafitinib
FLT3	1	Gilteritinib
KIT	1	Imatinib
NTRK1	1	Entrectinib, Larotrectinib
PDGFRB	1	Imatinib

## Discussion

Genetic analysis has greatly benefited the diagnosis and management of thyroid cancer in clinical practice. When it comes to detecting multiple genes, NGS technology has incomparable advantages (1). *BRAF*^V600E^ is the earliest established and most widely used molecular marker for PTC diagnosis (27). In MTC, various *RET* mutation sites are not only associated with tumor invasiveness but also serve as decisive indicators for whether or not to perform a prophylactic thyroidectomy (28, 29). The aim of this study is to provide a thorough understanding of common gene changes and their clinical significance in thyroid tumors using NGS.

In our cohort (*n* = 2844), 82% of the patients had gene changes detected. Cytology indeterminate nodules have been a diagnostic conundrum for both patients and doctors. With the help of NGS testing, 46 cytology indeterminate cases were diagnosed as malignant before surgery. Notably, four cases were false positives in NGS results. These nodules were considered benign according to postoperative pathology reports. The detailed gene variants are *KRAS*, *NRAS*, *EIF1AX,* and *BRAF*. The combination of cytology and NGS data resulted in significantly increased diagnostic sensitivity and decreased specificity. The two methods have no significant difference in negative predictive value (NPV). Both of their NPV values are quite low. This may be caused by the insufficiency of the NGS panel. We only used the 30-gene panel for this analysis. Further research should consider a larger NGS panel. The Youden Index is significantly lowered after the introduction of NGS. This may be caused by the selection bias of our cohort. NGS is normally prescribed in suspicious tumors with malignant manifestations. Again, a more thorough NGS panel needs to be involved to determine its diagnostic value. Nevertheless, NGS can be a useful preoperative diagnostic approach in addition to cytology, whose results should be read with caution to avoid false positive events.

*RET/PTC* and *NTRK3* have been widely reported to be associated with PTC diagnosis other than *BRAF^V600E^*. Notably, *CCDC6::RET* is correlated with a less aggressive PTC subtype and characteristics, while *NCOA4::RET* correlates with an aggressive phenotype of PTC (30). As for *NTRK3* fusion, it is an oncogenic driver in multiple solid tumors, including thyroid cancer (31). To the best of our knowledge, there has been no evidence regarding *RET/PTC* or *NTRK3* in benign thyroid tumors. In our cohort, 92 *RET/PTC* and 23 *NTRK3* were detected almost exclusively in PTC, apart from six (5.2%) cases of tumors with uncertain malignant potential. Our results indicate *RET/PTC* and *NTRK3* are highly correlated with PTC and can be used as diagnostic markers.

Our study, as well as other evidence found in prior researches (32) indicate that the coexistence of mutations including *BRAF*, *RAS*, *TERT*, and *TP53* generally predicts a poor prognosis for thyroid cancer. The prognostic value of the *BRAF^V600E^* mutation alone in PTC patients is still controversial. Numerous studies and meta-analyses have argued the association of the *BRAF*^V600E^ mutation with high-risk clinical pathological features (8, 9, 11, 18). However, in a large multicenter retrospective study, this association was no longer statistically significant after adjustment for clinical and histopathological features of aggressive thyroid tumors. The *BRAF*^V600E^ test did not increase the predictive value of PTC-related prognosis and mortality (12). In this study, *BRAF* positive patients have older age, smaller tumor size, lower vascular invasion, and higher tumor multifocality, showing no clear correlation between *BRAF^ V600E^* and the prognosis of PTC patients.

The* RET* proto-oncogene plays a central role in regulating cells through MAPK and PI3K-Akt signaling pathways (33). Germline mutation in the *RET* gene is dominant in almost all cases of hereditary MTC, while approximately 50% of sporadic MTC have somatic *RET* mutation. *RET* mutation can be further classified into different risk cohorts according to specific mutated codons (29). Although our study only tested mutations of MTC without the validation of blood, our results call attention to mutations at codons 918 and 634 in Chinese patients. When a co-mutation of *BRAF* and *RET* gene is detected, extra attention should be paid to detailed *RET* mutation site information. If* RET* mutation occurs elsewhere other than a high-risk site like codon 918, the thyroid tumor tends to present as a PTC with good biological behavior.

Previous studies have shown that mutation in the *TERT* promoter is associated with poor prognosis (34, 35). In our study, we have also confirmed that *TERT* mutated tumors exhibit more aggressive clinical features. *TERT* promoter mutation often co-exists with *BRAF* or *RAS* mutation in thyroid cancer. Patients with the coexistence of *TERT* promoter and *BRAF* mutations have significantly larger tumors, more extrathyroidal invasion, and more advanced TNM staging compared with patients with a *BRAF* mutations alone (10, 36). It is reported that the frequency of *TERT* promoter mutation is significantly increased in PDTC (40%) and ATC (50–70%) (32, 37, 38), and it tends to occur more often in senior patients. All these findings suggest that *TERT* promoter mutation may be an advanced molecular event which generates a synergistic effect with early molecular events to promote malignant transformation. Additional studies are thus urged to clarify the function and mechanism of *TERT* co-mutation events in the malignant progression of thyroid tumors.

Consensus has not been reached on whether or not to perform prophylactic neck dissection in thyroid cancer patients without clinically positive findings. In this study, we focus on those patients who were presumably cN0 but had pathology-confirmed central compartment lymph node metastasis after surgery. NGS data revealed that *BRAF* mutant patients have a significantly higher cervical lymph node metastasis rate than those without a *BRAF* mutation (28.24%, *P* = 0.003). Notably, patients with absolutely no identified gene mutation (*n* = 138) had a significantly lower rate of central lymph node metastasis compared with those who had at least one gene mutation detected (28.99% vs 37.97%, *P* = 0.039). We also investigate the correlation between certain gene mutations and lateral lymph node metastasis. Results show that patients with* TERT* (40.40% vs 25.14%, *P* = 0.001), *RET/PTC*1 (41.56% vs 25.23%, *P* = 0.001), and* CHEK2* (62.50% vs 25.56%, *P* = 0.047) mutations are more susceptible to lateral lymph node metastasis. Although the analysis may be affected by sample bias and surgeon experience, patients with certain virulence mutations tend to have neck lymph node metastasis and need to be taken into clinical consideration.

NGS sequencing of recurrent and aggressive thyroid tumors holds substantial clinical significance in guiding personalized targeted treatment approaches. However, extensive use of NGS will cause great waste, considering the majority of thyroid cancer can be controlled or even cured by surgery. PDTC and ATC, on the other hand, have poor prognoses and often in need of multiple treatment methods including surgery, targeted therapy, and immune therapy. NGS is undisputedly of great help in screening available targets for medication. In this study, *TERT*, *BRAF*, *TP53*, *RAS*, and *EIF1AX* mutations have a frequency of more than 10% in PDTC and ATC. Other genes that have been proven to be associated with tumor initiation or progression like *PIK3CA, ATM, PTEN,* and *CREBBP* are also present in PDTC and ATC with a mutation frequency higher than 5% (32). Therefore, NGS is essential for PDTC and ATC patients in seeking suitable targeted therapy.

Our study has some limitations that need to be stated. First of all, this is a retrospective study of the samples that were sequenced during a certain period of time. There’s no universal regulation or criteria on NGS detection at our institute. Doctors can prescribe NGS tests when they consider it necessary. Inevitably, selection bias exists in the study. Besides, the majority of our patient cohort has insufficient follow-up time; hence we have not recorded recurrence or death cases yet. Further observation of these patients is needed to support a valid survival analysis. Another limitation is that only 6% (170 cases) of the cohort is benign tumors. The disproportion of benign and malignant cases may impair our analysis. Moreover, the accurate information on somatic and germline *RET* mutations is available in this study, which tremendously restricts our investigation in MTC. In addition, the detection efficiency limited by NGS sequencing itself may lead to false-positive or false-negative results, for NGS is less sensitive in detecting a single mutation. Also, three different NGS panels differing in gene sizes were used in this study, which may affect the detection rate. Therefore, further studies with a more complete panel are warranted.

## Conclusion

In this study, we retrospectively analyzed NGS data of 2844 thyroid samples. Corresponding clinical features were investigated, and a mutation map was drawn for those patients. Our results reveal that NGS helps to diagnose cytologically indeterminate thyroid nodules before surgery. The *BRAF^ V600E^* mutation has no clear correlation with the prognosis of PTC patients, although *BRAF* mutant patients have a significantly higher cervical lymph node metastasis rate than those without a *BRAF* mutation. Mutations at codons 918 and 634 in the *RET* gene portend a very high incidence of MTC and a poor prognosis. Patients with *TERT*, *RET/PTC1,* and *CHEK2* mutations are more susceptible to lateral lymph node metastasis. The *TERT* promoter mutation shows very poor prognostic characteristics along with a high incidence of co-mutation, suggesting it may be a late molecular event that works with early molecular events in promoting malignant transformation.

## Supplementary Materials

Supplementary Tables

## Declaration of interest

The authors declare that there is no conflict of interest that could be perceived as prejudicing the impartiality of the research reported.

## Funding

This work was supported by the National Natural Science Foundation of Chinahttp://dx.doi.org/10.13039/501100001809 (81972501) and Shanghai Shenkang Hospital Development Center (SHDC22021201).
