# Supplementary material for: Real-world data analysis of next-generation sequencing and corresponding clinical characteristics in thyroid tumor
Source: Endocr Connect. 2024 Oct 9;13(11):e240301. doi: 10.1530/EC-24-0301 (PMC11562686; doi:10.1530/EC-24-0301)
Supplement: Supplementary Tables [file supplementary_tables.pdf]

Supplementary Table 1. Gene details of the three NGS panels used in this study

panel	Gene detail
30 genes	ALK、AKT1、*BRAF*、CHEK2、CTNNB1、EIF1AX、EZH1、FGFR1、FLT3、GNAS、H*RAS*、KIT、K*RAS*、N*RAS*、PIK3CA、PTEN、*RET*、SPOP、*TERT*、*TP53*、TSHR、ZNF148、NTRK1、CCDC6/*RET*、NCOA4/*RET*、PAX8/PPARG、ETV6/NTRK3
32 genes	ALK、AKT1、ATM、*BRAF*、CDKN2A、CDKN2B、CHEK2、CTNNB1、EIF1AX、EZH1、FGFR1、FGFR3、FLT3、GNAS、H*RAS*、KIT、K*RAS*、LRP1B、MAP2K1、MAP2K2、mTOR、N*RAS*、PDGFR-α、PIK3CA、PTEN、*RET*、SPOP、*TERT*、TET2、*TP53*、TSHR、ZNF148
88 genes	AGK、AKAP13、AKT1、ALK、APC、ATM、BANP、BCL2L11、*BRAF*、CDC27、CDH1、CDKN2A、CHEK2、CTNNB1、DICER1、EGFR、EIF1AX、EP300、ERBB2、ERBB4、EZH1、FARSB、FGFR1、FGFR2、FGFR3、FKBP15、GFPT1、GLIS3、GNAS、H*RAS*、IDH1、IDH2、IRF2BP2、KIT、K*RAS*、KRT20、MACF1、MED12、MEN1、MET、MKRN1、NCOR2、NF1、NOTCH1、N*RAS*、OFD1、PIK3CA、PTEN、RB1、*RET*、RNF213、ROS1、SLC5A5、SMAD4、SND1、STK11、*TERT*、TG、THADA、*TP53*、TSC2、TSHR、VCL、VHL、ZC3HAV1、ZNF148、CCDC6、NCOA4、KIF5B、PRKAR1A、GOLGA5、*RET*、TRIM24、HOOK3、ERC1、KTN1、TRIM27、PCM1、TRIM33、AKAP9、EML4、STRN、TPM3、NTRK1、TFG、PAX8、*BRAF*、ALK、NTRK3、PPARG

Supplementary Table 2. Genes with mutation frequency less than 0.5% in this study

gene	number	rate (%)
CHEK2	8	0.3
EZH1	7	0.2
SPOP	6	0.2
GNAS	4	0.1
ZNF148	4	0.1
EML4-NTRK3	3	0.1
CHEK2P2	3	0.1
CTNNB1	3	0.1
EIF1AX	3	0.1
ALK	2	<0.1
CDKN2A	2	<0.1
FGFR	2	<0.1
EZR-ROS1	1	<0.1
FLT3	1	<0.1
KIT	1	<0.1
NTRK1	1	<0.1
PDGFRB	1	<0.1
SQSTM1-NTRK3	1	<0.1

Supplementary Table 3. Detailed *RET* mutation which co-occurs with *BRAF* mutation

	Gene	mutational site	Exon	codon	amino acid	pathology
1	RET	c.1618A>G	8	540	Arg540Gly	PTC
2	RET	c.1618A>G	8	540	Arg540Gly	PTC
3	RET	c.1618A>G	8	540	Arg540Gly	PTC
4	RET	c.2094C>G	11	698	Asp698Glu	PTC
5	RET	c.2246G>C	12	749	Arg749Thr	PTC
6	RET	c.2326T>C	13	776	Phe776Leu	PTC
7	RET	c.2332G>A	13	778	Val778Ile	PTC
8	RET	c.2335C>A	13	779	Leu779Met	PTC
9	RET	c.2348A>G	13	783	Asn783Ser	PTC
10	RET	c.2441G>T	14	814	Gly814Val	PTC
11	RET	c.2462A>G	14	821	Lys821Arg	PTC
12	RET	c.2488G>A	14	830	Gly830Arg	PTC
13	RET	c.2488G>A	14	830	Gly830Arg	PTC
14	RET	c.2488G>A	14	830	Gly830Arg	PTC
15	RET	c.2753T>C	16	918	Met918Thr	PTC+MTC
16	RET	c.2753T>C	16	918	Met918Thr	PTC+MTC

Supplementary table 4. CHEK2 variants detected in this study

	Gene	mutational site	amino acid	significance
1	CHEK2	c.876del	Phe292fs	Pathogenic
2	CHEK2	c.1245dup	Lys416fs	Pathogenic
3	CHEK2	c.1245dup	Lys416fs	Pathogenic
4	CHEK2	c.1259A>C	Glu420Gly	Uncertain significance
5	CHEK2	c.1259A>C	Glu420Gly	Uncertain significance
6	CHEK2	c.417C>A	Tyr139Ter	Pathogenic
7	CHEK2	c.581G>A	Ser194Asn	Uncertain significance
8	CHEK2	c.581G>A	Ser194Asn	Uncertain significance
